# New Concepts in Median Nail Dystrophy, Onychomycosis, and Hand, Foot, and Mouth Disease Nail Pathology

**DOI:** 10.5402/2012/680163

**Published:** 2012-01-26

**Authors:** Nathan Y. Hoy, Alexander K. C. Leung, Andrei I. Metelitsa, Stewart Adams

**Affiliations:** ^1^Faculty of Medicine, University of Alberta, Edmonton, AB, Canada T6G 2R7; ^2^Department of Pediatrics, University of Calgary, Calgary, AB, Canada T2N 4N1; ^3^Department of Medicine, University of Calgary, Calgary, AB, Canada T2N 4N1

## Abstract

Nails are underutilized as diagnostic tools, despite being involved in many dermatologic conditions. This paper explores new concepts in the treatment of median nail dystrophy (MND), onychomycosis, and the nail pathology of hand, foot, and mouth disease (HFMD). A Pubmed database literature search was conducted for MND treatment, onychomycosis treatment, and HFMD nail pathology. Only papers published after January 2008 were reviewed. The results showed that 0.1% tacrolimus ointment can be an effective treatment for MND. Early studies on laser therapy indicate that it is a safe and efficacious treatment option for onychomycosis, compared to conventional oral antifungal agents. Vicks VapoRub (The Proctor & Gamble Company, Cincinnati, OH) is effective against onychomycosis and is a reasonable option in patients who choose to forgo conventional treatments. Lastly, there is evidence to support a correlation between HFMD and onychomadesis.

## 1. Introduction

Nails are often underutilized as a diagnostic tool in dermatology, despite being involved in a wide variety of dermatologic conditions. New ideas in pathophysiology, advances in diagnostic and management techniques, and innovations in treatment are continuously evolving in the field of nail disease. In this paper, we aim to shed light on some of these fascinating developments with respect to the treatment of median nail dystrophy (MND), onychomycosis, and the nail pathology of hand, foot, and mouth disease (HFMD). 

At a basic level, the nail unit is composed of a matrix on a bed, surrounded by skin. The distal nail matrix is called the lunula, which is the half-moon shape at the base of the nail, and is responsible for the production of the ventral nail plate. Melanocytes are also contained within the nail matrix and are usually quiescent but may become active and impart pigmentation to the keratinocytes in the nail plate. The nail plate overlies the nail bed, which contains blood vessels and nerves. Skin surrounding the nail plate composes the perionychium, which can be further divided into proximal and lateral nail folds and the hyponychium, the area beneath the free edge of the nail [1].

Production of the nail plate is continuous from embryonic development until death. The mean growth rate of fingernails and toenails per month is 3 mm and 1 mm, respectively, implying approximately 4–6 months to completely regenerate a fingernail or 8–12 months to replace a toenail. Nail growth is linked to a number of factors, such as age, presence of systemic and localized diseases, and medications [1, 2].

## 2. Methods

Using the Pubmed database, the literature was searched in three groups using the following terms: Group 1, “median nail dystrophy”, “median canaliform dystrophy of Heller”, and “treatment”; Group 2, “onychomycosis”, “treatment”, and “laser”; and Group 3, “hand foot mouth” and “nail.” As well, Google searches were carried out using the same terms. Only papers published after January 2008 were reviewed since this paper aims to provide an overview of the latest literature. Articles published before this time period were also used to provide background information.

## 3. Results and Discussion

### 3.1. Median Nail Dystrophy

Median nail dystrophy (MND), also known as dystrophia unguium mediana canaliformis or median canaliform dystrophy of Heller, is characterized by a paramedian canal or split in the nail plate of one or more nails [3]. Small cracks or fissures that extend laterally from the central canal or split toward the nail edge give the appearance of an inverted fir tree or Christmas tree. The condition is usually symmetrical and most often affects the thumbs, although other fingers or toes may be involved [4].

The pathophysiology of MND is still unknown. Presumably, the condition results from a temporary defect in the matrix that interferes with nail formation [4]. Trauma has been implicated as a causative factor [4, 5]. Habitual picking of the nail base may be responsible for some cases [4]. Two cases of MND have been reported following the habitual use of personal digital assistants for 4 to 8 months [5]. The MND resolved in a few months after personal digital assistant usage was discontinued [5]. It has been shown that some patients on oral isotretinoin may develop MND, with subsequent resolution upon discontinuation of the medication [6, 7]. Rarely, familial occurrences of MND have been reported [8].

Treatment of MND remains a difficult undertaking, as no therapy has been shown to be consistently successful. Most treatments revolve around injecting medications, such as triamcinolone acetonide, into the dystrophic nail [9]. Injections are difficult to tolerate and result in numerous adverse effects for many patients. Furthermore, the efficacy of such treatments is quite variable. The most recently reported treatment for MND involves topical application of 0.1% tacrolimus ointment once daily without occlusion [4]. Kim et al. reported a 19-year-old man with MND affecting both thumbnails [4]. He was treated with a topical corticosteroid, applied around the proximal nail fold twice a day, with no remarkable changes after 4 weeks. He was then treated with 0.1% tacrolimus ointment, applied on the proximal folds of both thumbnails without occlusion every night. After 4 months, significant clinical improvement of both thumbnails was observed. It is speculated that calcineurin inhibitors are an effective treatment due to their interference with the inflammatory component of MND [4]. Additional evidence for the effectiveness of tacrolimus in treating nail dystrophy is seen in its successful treatment of nail dystrophy associated with lichen planus [10]. None of the aforementioned reports documented side effects associated with the application of tacrolimus ointment to the affected nail, suggesting that it is a safe and effective therapeutic option for MND. However, further randomized controlled trials are necessary to provide supporting evidence for this new therapy.

### 3.2. Onychomycosis

Onychomycosis is a fungal infection of the nail, presenting with nail discoloration, thickening, irritation, pain, and detachment of the nail plate. A number of organisms are responsible, including dermatophytes, non-dermatophyte molds, and Candida. It is an exceptionally common problem, with studies estimating its prevalence around 14% in the general population [11]. Dermatophytes such as *Trichophyton rubrum* and *Trichophyton mentagrophytes* account for 80 to 90% of all cases [12]. Nondermatophyte molds that can cause onychomycosis include *Acremonium* species, *Alternaria* species, *Aspergillus* species, *Fusarium* species, *Scytalidium* species, and *Scopulariopsis* species [12]. *Candida albicans* accounts for approximately 70% of onychomycosis caused by yeasts. Predisposing factors include hyperhidrosis, wearing occlusive shoes, participation in sports, use of commercial swimming pools, contact with sources of fungal infection, nail trauma, immunodeficiency, diabetes mellitus, and old age.

Current treatment options are less than ideal with respect to efficacy, side effects, and convenience. Terbinafine has long been the most effective oral antifungal medication for dermatophytic infections. Studies have shown clearance rates of 76% over 12 weeks [13], but terbinafine remains a non-ideal modality due to its potential for hepatotoxicity, drug interactions, and ineffectiveness against nondermatophytes [14].

Regarding other oral antifungal medications, Griseofulvin was the first oral antifungal agent approved for use in the US. It has significantly more drug interactions and adverse events and is less effective than azoles and terbinafine [15], essentially removing it from the current dermatologist's treatment options. Numerous studies have established terbinafine to be superior to itraconazole in the treatment of dermatophytic onychomycosis [16]. However, itraconazole has broader coverage for Candida and non-dermatophytic molds. As a result, azoles are generally used as first line in the treatment of these less common infections. Unlike terbinafine, azoles are fungistatic, as well as having the potential for more adverse reactions and drug interactions, making it difficult to utilize in patients with comorbidities and polypharmacy [17]. The difficulties with tolerability in systemic itraconazole for onychomycosis were demonstrated in a study by Gupta et al., where 23% of patients (*n* = 1063) on 200 mg/day of itraconazole experienced adverse events such as headache, rash, or gastrointestinal symptoms and 7% had to stop treatment due to these adverse events [18].

The need for a therapeutic modality with minimal systemic side effects, a reasonable duration of therapy, and an ability to deliver treatment to a confined area has spurred the recent development of several new treatments. Laser therapy has the potential to be an ideal treatment option with respect to its ability to deliver a high concentration of energy in a small area. This limits systemic side effects and enhances its ability to penetrate deeper into the nail plate, eradicating all residual fungal elements. The theoretical mechanism of action of infrared laser is heating fungal cells to the point of structural and functional impairment in their infectious activity. Early evidence both *in vitro* and *in vivo* has shown promising results for the efficacy of lasers in the treatment of onychomycosis (Table 1). 

The Q-switched Nd:YAG laser with 532-nm and 1064-nm wavelengths is capable of inhibiting the growth of *T. rubrum in vitro*. The inhibitory effect is likely due to more than simple nonspecific thermal damage [19]. Although the 532-nm setting is well absorbed by red pigment in canthomegnin in *T. rubrum*, the effective inhibition of the fungus also requires very short pulses of 532-nm wavelength that generate mechanical damage in the irradiated fungal colony. The 1064-nm setting is beyond the absorption spectrum of xanthomegnin, but its effectiveness is postulated to be due to another absorbing chromophore, such as melanin, present in the fungal cell wall [19]. The effectiveness of Q-switched Nd:YAG laser with 532-nm and 1064-nm wavelengths has yet to be tested *in vivo*.

Manevitch et al. obtained 99 nail cuttings from patients with onychomycosis caused by *T. rubrum* [20]. Nail cuttings with positive fungal growth underwent femtosecond infrared titanium sapphire laser irradiation using increasing laser intensities with the focus scanned throughout the whole thickness of the nail specimen. The authors found that femtosecond laser fluencies of 7 × 10^31^ photons m^−2 ^s^−1^ or above successfully inhibited the growth of the fungus in all samples examined. However, laser intensities above 1.7 × 10^32^ photons m^−2 ^s^−1^ damaged the structure of the nail plate. These findings suggest that *T. rubrum* onychomycosis can be treated by femtosecond laser technology.

Clinically, Kozarev and Mitrovica demonstrated a 100% clearance rate in 42 nails with onychomycosis over 12 months, with minimal side effects, using a novel 25-millisecond pulsed 1064-nm Nd:YAG laser [21]. More recently, Hochman successfully treated 7 out of 8 onychomycosis patients with a 0.65-millisecond pulsed Nd:YAG 1064-nm laser over the course of two to three treatments spaced at least 3 weeks apart, without any significant side effects [22]. The results from this study may have been confounded by the prophylactic daily application of antifungal cream on each toe to prevent recolonization.

The study by Landsman et al. was the first one to stratify the patient population by severity [23]. They showed a unique dual-wavelength near-infrared diode laser with 830-nm and 970-nm to be effective against onychomycosis of all severities. Out of the 26 toenails treated (ten mild, seven moderate, and nine severe), 22 (85%) displayed improvement after 180 days of followup and 4 rounds of treatment over the course of 4 months. Sixty five percent showed at least 3 mm, and 26% showed at least 4 mm of clear nail growth. Of the 16 toes with moderate to severe involvement, ten (63%) improved, as shown by clear nail growth of at least 3 mm.

As demonstrated by these studies, laser therapy has the potential to be an ideal treatment modality for onychomycosis. Laser therapy is clean, efficient, effective, safe, and tolerable. It delivers an effective therapeutic dose to a limited area, with no potential for drug interactions. Exact treatment parameters have yet to be established, but as the number of clinical trials increases, there is the potential to establish consensus guidelines. The greatest impediment to the mass implementation of laser therapy is the cost of the laser unit itself.

Another treatment option that lacks the side effects of oral antifungal medications is Vicks VapoRub (The Proctor & Gamble Company, Cincinnati, OH) [24]. This treatment has been discussed in the layman literature but, until recently, has never been studied scientifically. Numerous ingredients in Vicks VapoRub have been studied *in vitro*, including thymol, menthol, camphor, and oil of Eucalyptus, and have demonstrated efficacy against dermatophytes [24].

In a recent study, Derby et al. treated 18 patients with clinical onychomycosis that was evident on at least one great toenail with Vicks VapoRub applied to the affected nails daily [24]. Patients were followed at intervals of 4, 8, 12, 36, and 48 weeks. Digital photographs were obtained during initial and follow-up visits. Primary outcome measures were mycological cure at 48 weeks and clinical cure through subjective assessment of appearance and quantifiable change in the area of affected nail by digital photography analysis. Fifteen (83%) patients showed a positive treatment effect; 5 (27.8%) had a mycological cure and clinical cure at 48 weeks; 10 (55.6%) had partial clearance; 3 (16.7%) showed no change. Despite not everyone demonstrating improvement, all 18 participants were “satisfied” or “very satisfied” with the nail appearance, based on a 5-point Likert scale survey. The limitations of the study include its small sample size, lack of control group, and variability in the infectious agent. Although there is insufficient evidence to recommend it as a first line treatment, this study demonstrates that there is some efficacy for Vicks VapoRub. Given its very localized effects and safety profile, it is not unreasonable to recommend this treatment in patients with contraindications to, lack of access to, or who refuse conventional options.

### 3.3. Nail Pathology in Hand, Foot, and Mouth Disease

HFMD is usually caused by coxsackievirus A 16. Less commonly, it is caused by coxsackievirus A4, A5, A6, A7, A9, A10, A24, B2 to B5, enterovirus 71, and echoviruses [25]. All are RNA viruses and they spread by fecal-oral and respiratory routes. Spread to other family members commonly occurs.

HFMD is characterized by vesicular stomatitis and cutaneous lesions on the palms and soles. The disease has an incubation period of 3 to 6 days [26]. There is usually a mild prodrome consisting of low-grade fever, anorexia, sore mouth, and malaise. Children younger than 10 years are most commonly affected. Oral lesions occur chiefly on the anterior buccal mucosa and tongue, where the vesicular surfaces are eroded rapidly, leaving ulcers with erythematous borders [26]. The lesions on the palms and soles are papules or vesicles on a surrounding zone of erythema. Less commonly, the dorsal or lateral surfaces of the hands and feet may also been affected. Involvement of the buttocks is common, but typically there is a lack of vesiculation [26]. The eruptions are nonpruritic and usually resolve without crusting [25]. The association between HFMD and onychomadesis was first proposed by Clementz and Mancini in 2000 and Bernier et al. in 2001 [27, 28]. Onychomadesis is the spontaneous separation of the nail plate from the matrix starting at the proximal edge and is the result of the temporary cessation of nail formation [4]. Recently, authors in Spain and Finland have brought forth evidence to solidify the association between these conditions [29–33].

A study of an outbreak of HFMD in daycare centers and schools during the fall of 2008 in Finland led to one characteristic feature being found amongst the infected population: shedding of the nail plate approximately 1-2 months after the onset of classic HFMD symptoms [29]. Redondo et al. documented a similar onychomadesis observation during an HFMD outbreak in Valencia, Spain in the winter of 2008 [30]. Fifteen children and one adult presented with nail changes consistent with onychomadesis, a mean of 6 weeks after the clinical diagnosis of HFMD. The nail changes were temporary with spontaneous normal regrowth in 1–4 months.

Three other studies of HFMD epidemics revealed similar associations between onychomadesis and HFMD [31–33]. One study found that out of 221 patients with onychomadesis presenting during an HFMD outbreak, 134 (61%) had HFMD [33]. The median duration between the clinical diagnosis of HFMD and observation of onychomadesis was 39 days. Of note, this study represents the first identification of the etiologic agents responsible for onychomadesis seen with HFMD [33]. Bernier et al. initially proposed that multiple enterovirus strains were capable of causing the onychomadesis, and the findings of this study support that hypothesis [28].

The potential causal relationship between enterovirus causing HFMD and onychomadesis has never been proven and is only a well-defined temporal relationship. One potential explanation for the relationship between the two, aside from enterovirus causing onychomadesis, is intensive hygienic measures during HFMD outbreaks leading to maceration and a local environment favoring *Candida *infection and allergic contact dermatitis, both of which can cause onychomadesis [34]. Haneke suggests that proximal nail bed inflammation in HFMD may cause the nail dystrophy, which is a more likely explanation of the association [34]. The author also notes that viral determination from stool and pharynx samples takes 1–3 weeks following the diagnosis of onychomadesis and thus, between seven and nine weeks after the HFMD infection [34]. To further compound this issue, HFMD is a self-resolving condition that has a natural time course of approximately one week. This severely limits the ability to implicate the virus causing HFMD as the de facto causative agent of onychomadesis following HFMD. If another HFMD outbreak were to occur, it may be possible to take weekly swabs from under the nails of HFMD- afflicted patients and send them for viral analyses. This would allow for the establishment of the nail microbial milieu and comparison between those patients who develop onychomadesis and those who do not, to determine if the virus causing HFMD is present in the dystrophic nail.

## 4. Conclusion

Rapid advances have been made in the field of nail dermatology as demonstrated through the examples of MND, onychomycosis, and HFMD. It has been shown that MND can be effectively treated by daily applications of 0.1% tacrolimus ointment [4]. Current treatment options, including corticosteroid injections, are highly uncomfortable for the patient and have not been shown to be consistently effective [9]. Lasers are a safe and efficacious treatment option for onychomycosis and have the potential to become part of the first line therapies with more evidence [19–23]. Compared to oral antifungal agents, laser treatments have minimal systemic side effects, are more effective, and have no risk for drug interactions. Vicks VapoRub has also been shown to be effective in the treatment of onychomycosis without side effects and is a reasonable option in patients who choose to forgo conventional treatments [24]. Lastly, there is new evidence to support a correlation between HFMD and onychomadesis [29–33]. This is useful when advising patients what to expect following an episode of HFMD or if onychomadesis is the initial presentation, to seek a history of HFMD as a potential etiology. Given the vast number of skin diseases with nail manifestations and number of primary nail conditions, it is prudent to have a firm understanding of how to manage and treat nail pathology using therapies supported by the latest scientific evidence.

## Figures and Tables

**Table 1:** Summary of *in vitro* and *in vivo* studies demonstrating efficacy of laser therapy in the treatment of onychomycosis.

Study	*In vitro or in vivo*	Laser model	Outcome
Vural et al. [19]	*In vitro*	Q-switched Nd:YAG laser 532-nm and 1064-nm wavelengths	Inhibition of fungal colony growth
Manevitch et al. [20]	*In vitro*	Femtosecond infrared titanium sapphire	Complete clearance of *T. rubrum *infected nail clippings after 4 weeks
Kozarev and Mitrovica [21]	*In vivo*	25-millisecond pulsed Nd:YAG laser 1064 nm wavelength	100% clearance in 42 nails after 3 weeks of treatment
Hochman [22]	*In vivo*	0.65-millisecond pulsed Nd:YAG laser 1064 nm	7/8 cleared after 9 weeks of treatment
Landsman et al. [23]	*In vivo*	Dual-wavelength near-infrared diode laser with 830-nm and 970-nm wavelengths	22/26 demonstrated improvement after 4 months of treatment
